# Stability Loss Analysis for Thin-Walled Shells with Elliptical Cross-Sectional Area

**DOI:** 10.3390/ma14195636

**Published:** 2021-09-28

**Authors:** Ján Kostka, Jozef Bocko, Peter Frankovský, Ingrid Delyová, Tomáš Kula, Patrik Varga

**Affiliations:** 1 Department of Applied Mechanics and Mechanical Engineering, Faculty of Mechanical Engineering, Technical University of Košice, 042 00 Košice, Slovakia; jan.kostka@tuke.sk (J.K.); jozef.bocko@tuke.sk (J.B.); ingrid.delyova@tuke.sk (I.D.); tomas.kula@tuke.sk (T.K.); 2 Department of Biomedical Engineering and Measurement, Faculty of Mechanical Engineering, Technical University of Košice, 042 00 Košice, Slovakia; patrik.varga@tuke.sk

**Keywords:** thin-walled shells, stability loss, tensile test, FEM

## Abstract

The aim of the scientific contribution is to point out the possibility of applicability of cylindrical shells with a constant elliptical cross-sectional shape for stability loss analysis. The solution to the problem consists of two approaches. The first approach is the experimental measurement of critical force levels, where the work also describes the method of production of the sample and jigs that cause the desired elliptical shape. The second approach is solving the problem in the use of numerical methods—the finite strip method together with the finite element method.

## 1. Introduction

In the introduction, it should be emphasized that the problem of loss of stability for thin-walled shells with an elliptical cross-sectional area has gradually evolved over the past and present centuries. With the advent of more innovative experimental and numerical methods, more accurate results can be achieved. Below, in the article in the historical and current overview of the development of the loss of stability survey, the researched issue is described with individual gradual solutions.

One of the first works that dealt with the issue of shell elements with elliptical cross-section is a work published by the author Brown in 1936 [1]. His work describes and conducts the dependencies between stress and strain for shell elements with an elliptical cross-section, which are subjected to internal compressive loads. During this period, many scientists and researchers have focused their attention primarily on shells with a circular cross-section. The obvious reason for this is that structural elements of this type occur very frequently.

The rapid development of society, which occurred in the late 40s of the 20th century, brought many problems, especially in the field of aviation. One of the unexplored areas of aviation was the increase in the speed of aircraft, which approached the speed of sound or overcoming it. A serious problem that occurred was the effect of compressive stress on the leading edges of the fighter wings. This type of problem connected with the nose of the wing, whose stability is affected by the changing curvature of the shell, was solved by Marguerre in 1951 [2].

In the study [3], the authors present the derived knowledge for the solution of cylindrical shells using the energy method for the problem of oval or elliptical cylindrical shells. The authors consider precisely defined geometric dimensions of the elliptical cross-section of the shell and defined boundary conditions. Another important contribution for the given issue of elliptical shells was published by the same authors in [4]. This contribution is mainly based on a dissertation thesis by Chen published in 1964 [5]. In a subsequent paper [6], the authors describe energy expressions and related differential equations for non-circular cylindrical shells that are analogous to the relationships with Donnell derived for circular cylindrical shells. In 1966, a large symposium was held to address this issue, where Kempner and Chen presented their achievements to date, which are reported in the paper [7]. One of the other authors who dealt with the problem of stability loss in elliptical shells was Hutchinson, who presented his findings in an extensive work [8] in 1968. The study [9], brings an extension of the problem in the nonlinear area. The work [10], deals with the problem of the loss of stability of oval shells, examining the induced state of the shell due to fit. For this case, the authors have chosen the fixed boundary condition of the shell and in the development of this issue they define a relatively precise state of pre-stress in the boundary condition. To solve the stability equations, they use the Fourier method with a combination of higher order differential techniques. Subsequently, the authors in [11], experimentally verify the developed theory described in [10]. Their findings indicate that there is sufficient similarity in the negative impact of the geometric imperfections of the shell surface, as in the case of circular cylindrical shells. Other authors who dealt with this negative phenomenon (the effect of geometric imperfections) published their findings in [12], addressing the dependence and the effect of magnitude of eccentricity on the resulting value of the critical force. An interesting contribution is the work of the authors [13], where they use a laser interferometer to determine the magnitude of the deformation of the casing caused by the loss of stability.

The experimental results obtained are compared on the basis of the theory derived by Bresse and MacAlpine. 1974 is a turning point for authors [14]. In the work they published, they extensively described the condition of the elliptical shell, which is subjected to a clear bending load and a combination of axial compressive load and bending. The first and second order stability equations are derived from Donnell’s assumptions because they have proven to be sufficiently accurate. A subsequent important finding is the fact that some types of elliptical shells are able to carry higher or lower load values than the reference circular cylindrical shell. However, the known fact has been confirmed that if the shells of both types of elliptical shells contain initial geometric imperfections, this has a major effect on the resulting shells bearing capacity, as in the case of a circular cylindrical shell. The greater the geometric imperfection the lower the bearing capacity of shell is. The authors thoroughly describe the condition of the oval shell under the influence of compressive load and axial-symmetric bending in [15]. In 1994, the authors [16] solved the issue of laminate elliptical shells. The subject of their research was the change of the natural frequency of the vibration, based on the different method of laminating. The equations of motion were derived on the basis of the Lagrangian (Hamilton) principle. The author of the work [17] deals with the problem of loss of stability of an elliptical cylindrical shell, which is created with the composite materials. A large publication is the author’s work [18], which deals with the use of various elliptical cylindrical shells for general construction use. Two methods of loading were considered in the experimental measurements. Geometric imperfections, material properties and recorded history of loading cycles are considered as input parameters that negatively affect the resulting shell strength. The experimentally determined values were subsequently verified using the numerical calculation program ABAQUS^®^. Another author who used numerical methods to solve stability loss of elliptical shell due to local loss of shell surface was [19]. In the past, there were not many options for implementing the advantages of elliptical shell elements into the design. The problem is that there was no exact design-defined procedure for these types of structural elements. For this reason, the authors in [20] describe and implement the overall design of elliptical shells according to the valid Eurocode 3, BS 5950-1, AISC 360-05, and AS 4100 (the validity of these approaching applies to the then current and valid standards, year 2008). The author’s text [21] describes the procedure for determining the shape of the collapsed shell surface, while his theoretical knowledge is based on the presented works of the authors mentioned above. An interesting contribution is the work of authors [22] in the field of biomedical engineering. In their study, they examine the effect of the cross-sectional shape of an artery and the relationship between an increase in blood pressure and an artery collapse. At present, the author deals with the issue of geometric imperfections of the elliptical shell surface in [23]. Subsequent interesting work from the discussed field is presented by the authors [24,25].

The aim of this paper is to present experimental and numerical procedures for solving the stability problem of axially loaded thin-walled shells with elliptical cross-sections. The first part of the experimental measurements consists of determining the wall thickness of the shell used and identifying the material properties. The results of these measurements served as input data for the numerical computations. The second group of measurements was oriented toward the measurement of critical axial forces for shells with different degrees of eccentricity of the elliptical cross-section. For the numerical simulation performed in the linear domain, two methods were used: the finite element method and the finite strip method.

## 2. Stability Concept of Thin-Walled Shells, Proposal of Fixture with Elliptical Cross-Section, and Manufacturing Shell Specimen

**Figure 1:**
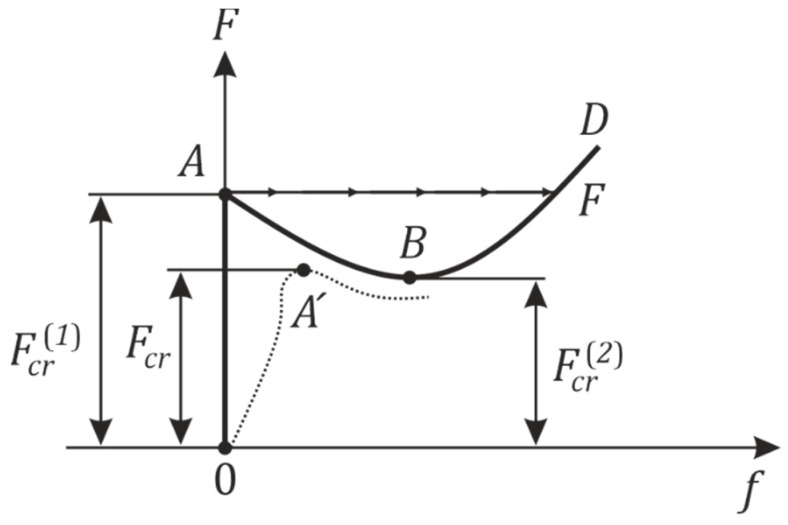
The shell element diagram for loss of stability.

Consider a theoretically perfect case of a shell element with both ends which are simply supported and on which acts a uniform axial compressive load. Its loss of stability diagram can then be represented by Figure 1.

The OABD curve represents a perfect case of a shell element. The load F is applied statically so that the initial equilibrium configuration of the shell is in the membrane state of stress f=0. The equilibrium part of the OABD consists of growing branches OA and BD, which correspond to equilibrium stability states, and the descending branch AB corresponds to an unstable equilibrium configuration. Point A on the OABD curve can also be interpreted as a bifurcation point. The peculiarity of shell elements, in contrast to other thin-walled structural elements, is the assumption that no new adjacent deformed stable equilibrium configurations, located in the infinite proximity of point *A*, are created [26,27,28]. Stable deformed equilibrium configurations are defined from the originals at finite distances on the BD branch. Thus, the transition from the original equilibrium configuration on the OA branch to the new stable deformed equilibrium configuration on the BD branch is performed by jumping from the steady state A through the static unstable state given by the branch AB to the new steady state F on the branch BD. Such a phenomenon with a corresponding jump at point F is referred to as a loss of shell stability. In the case of a real shell element, the stability loss diagram can then be described as follows. The area of shell instability for a given real shell is shown by the dashed line in Figure 1. It can be seen, that due to the initial imperfections, the actual shell begins to deviate from the original equilibrium configuration at the beginning of the load. Therefore, it is not possible to assume that this shell is in the original membrane state of stress. The branch OA, during the increase of the load does not coincide with the axis F. Therefore, the transition from the original steady state to the deformed one also occurs by a jump, but at the level of the limit point A′ [26,29,30,31]. It follows from the above that there are three different critical load values that characterize the properties of thin-walled shells when a loss of stability occurs:

- $F_{cr}^{\left(1\right)}$ is the upper critical load and can be defined as the largest load to which the original shell equilibrium configuration remains stable with respect to minimal imperfections;
- $F_{cr}^{\left(2\right)}$ is the upper critical load and can be defined as the lowest load to which the original shell equilibrium configuration of the shell remains stable with respect to minimal imperfections;
- $F_{cr}$ is the critical load of the real shell element, referred to as the buckling load, and can be defined as a load with a certain value at which the deflection of the actual shell surface occurs, i.e., such a critical load value for which the original equilibrium state of the shell ceases to be stable [26].

**Figure 2:**
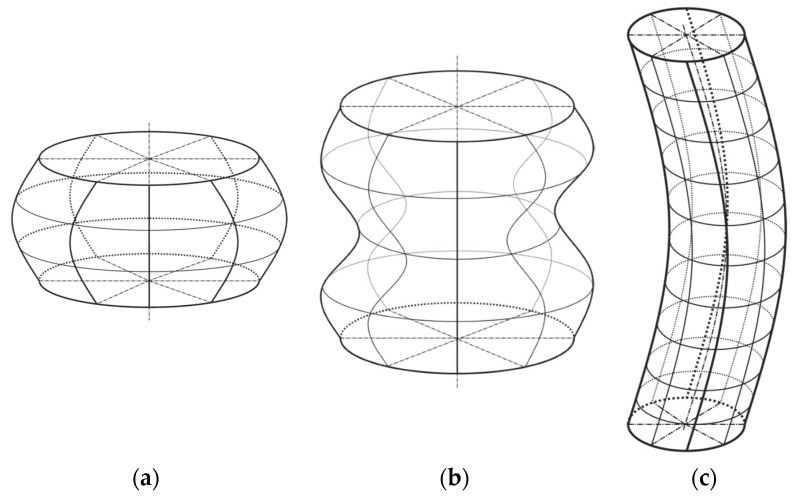
Collapsed shapes of: (**a**)—short; (**b**)—medium; (**c**)—long shell classes.

Cylindrical thin-walled shell elements can be divided into three classes in terms of their length and radius ratio. Each of these classes has its own specifics of establishing the loss of stability under the influence of axial compressive load. The first class represents shells of short lengths. The onset of loss of stability is accompanied by the phenomenon where the collapse of the casing is formed mostly by only one sinusoidal half-wave in the axial direction. The second class represents shells of medium lengths. The onset of loss of stability can be assumed in two variants. The collapse of the shell can be caused by a local loss of stability induced on the shell surface as a result of various geometric and material imperfections, or by a global loss of stability as a result of loading processes and the way the structure as a whole is laid. From the point of view of the study of loss of stability, this class of shells is the most difficult to solve. The third class represents long shells. The establishment of a loss of stability is accompanied by the phenomenon in which the shell behaves as a buckling of a long rod. The induced collapse of the shell then generally looks like a bend in the radial direction without damaging the cross-sectional area of the surface [32]. All three types are shown in Figure 2 and mathematical division into individual classes is defined by the form

(1) $$Z=\frac{L^{2}}{Rh}\sqrt{\left(1-\mu^{2}\right)}$$

where, Z represents the dimensionless form factor which represents the measure of the ratio of the shell length to its radius, L represents the shell length, R represents the shell radius, h represents the shell surface thickness, and μ represents the Poisson’s ratio [32].

If the magnitude is acquired

(2) Z<2.85

then the shell represents a short length class, and if

(3) Z>2.86

then the shell represents a class of medium and long lengths.

The field of investigation for the loss of stability of thin-walled shells focuses on cylindrical shells with an elliptical cross-section. The task is to determine the influence of the elliptical cross-sectional area on the achieved critical force value. Four fixtures are designed to provide the desired elliptical cross-section along the entire length of the test specimen. The size of the ellipses examined was defined by a parameter referred to as eccentricity e. The relation determining e is defined as

(4) $$e=\sqrt{1-\frac{b^{2}}{a^{2}}}$$

where, the designations a, b represent the ellipse arms.

**Figure 3:**
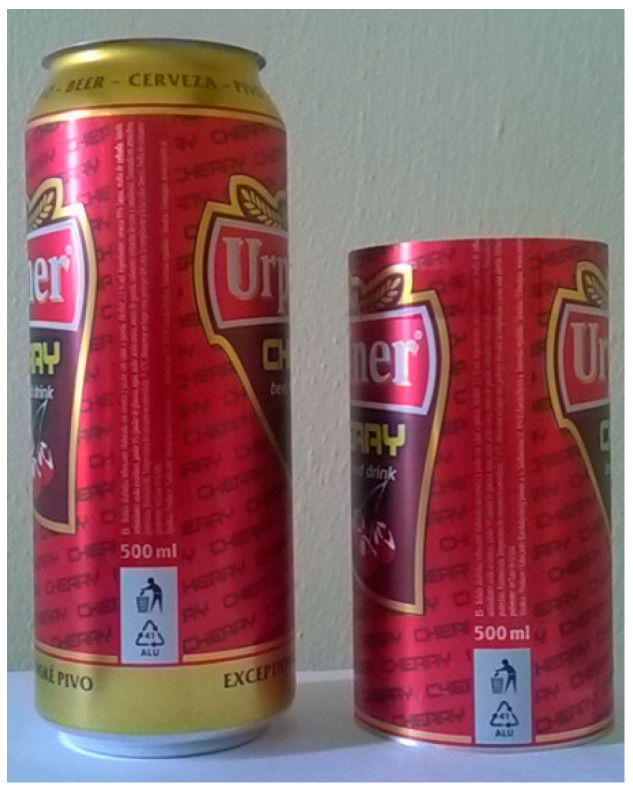
Can and manufactured specimen.

Thin-walled shell elements are specific in that they can carry significantly higher loads than other thin-walled elements. In order to minimize this positive property of the shells, a can designed for storing carbonated beverages with a volume of 0.5 L, was chosen for the production of test specimens. In order to make a correct test specimen from the can, it was necessary to remove both ends of the can in precisely defined places. These points are located behind the can rounding, where the shell has constant diameter and cross-sectional surface thickness is identical, Figure 3.

The sample thus produced can be considered as a thin-walled shell with a circular cross-section. By applying mechanical fixtures, it is possible to create the desired elliptical cross-sectional areas. The geometric dimensions of the test specimen are:

- Total length of specimen is $L_{c}=120\;\mathrm{mm}$,
- Length of surface working part is *L* = 100 mm,
- Specimen radius is R=33 mm.

**Figure 4:**
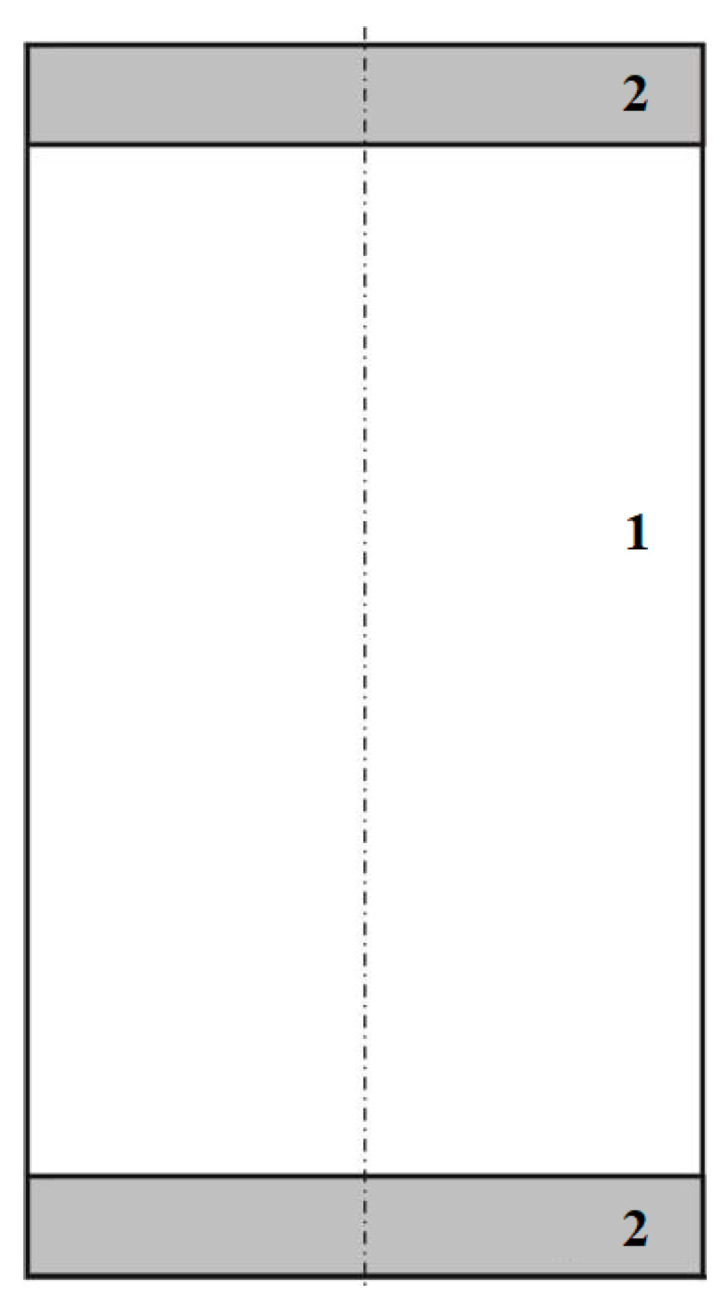
Schematically representation of surface test specimen.

Figure 4 shows a schematic representation of a test specimen with areas indicated. Label 1 represents the functional part of the specimen surface that is subject to stability loss investigation, and Label 2 represents the part of the surface that attaches to mechanical fixtures which are causing elliptical cross-section of specimens.

### 2.1. Static Tensile Test for Determining Material Parameters of Aluminium Alloy

**Figure 5:**
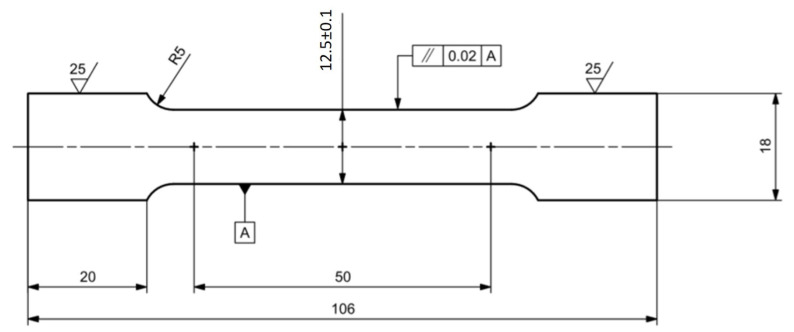
Proposed specimen for static tensile test, all dimensions are in mm.

The test specimens were made from a can intended for storing carbonated beverages. As is known, such cans are mostly made of deep-drawn aluminum alloy. In order to make the numerical calculation as accurate as possible and at the same time to correspond as closely as possible to the actual state of the can material affected by the production technology, it was decided to obtain basic material parameters directly from the can and not to apply aluminum alloy material data from its material sheet. Based on currently valid standard [33], a variant of the specimen intended for the tensile test was proposed in Figure 5. For validation process of static tensile test, it is necessary that the produced samples meet geometric and dimensional tolerances. The main dimension to be monitored is the functional width of the test specimen 12.5±0.1 mm, which is closely connected with the functional length dimension 50 mm.

**Figure 6:**
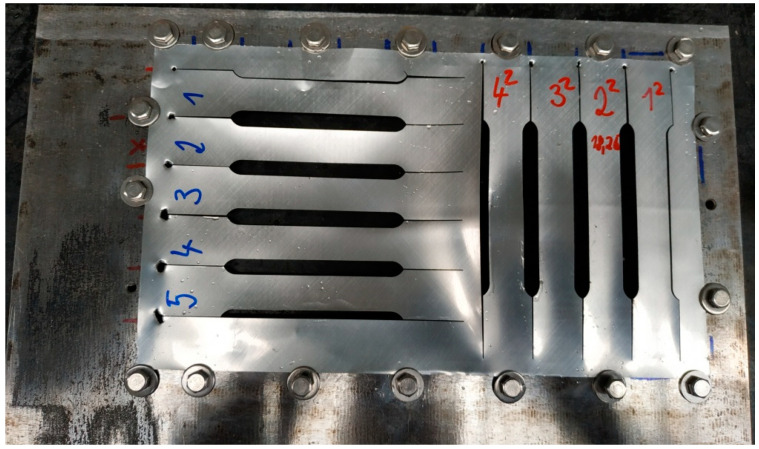
Manufactured specimen for static tensile test, blue mark = axial orientation of material fiber, red mark = radial orientation of material fiber.

The production of specimen was made by a wire cutter. Due to the manufacturing process, it was necessary to develop a mechanical fixture, whose function is fixing developed can surface, Figure 6.

**Figure 7:**
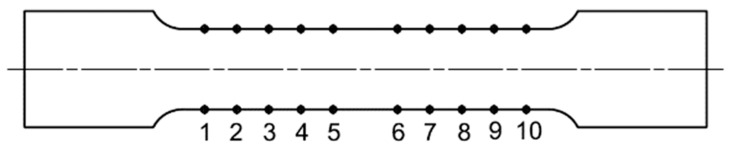
Selected locations for control measurements along the functional length dimension of specimen.

Cutting the desired shape of the test specimen into the developed can surface caused the release of residual stresses in the materials that were as a result of the can manufacturing technology process. This factor negatively affected the functional dimensions of the manufactured specimen, which had to be followed in order for the samples to be used for the static tensile test. Subsequent modification of the fixing of the developed can surface in the mechanical fixture and adjustment of the cutting parameters of the production tool significantly eliminated this negative factor for specimens which are marked by red color. For specimens marked by blue color, this negative effect was only partially eliminated, confirming the performed control measurement of the produced specimen. A control measurement of the functional width dimension specimen was performed at 10 locations. The control measurement points are shown in Figure 7. In this way, specimens were produced whose fiber orientation of the material is in the axial direction of the can, which means that the fiber orientation is along the length of the can, and in the radial direction of the can, meaning that the fiber orientation is circumferential direction. Introduced color marking, divides individual types of manufactured specimen, Figure 6. Specimens with material fibers having axial orientation are marked in blue and specimen with material fibers having a radial orientation are marked in red. The reason for the production of these two specimen variants is the assumed anisotropic state of the can material.

### 2.2. Proposed Numerical Solution for Finite Strip Method

Section 2.2 deals with the theoretical background of FSM and the creation of stiffness matrices, transformation matrices and transformations from a local coordinate system to a global one.

**Figure 8:**
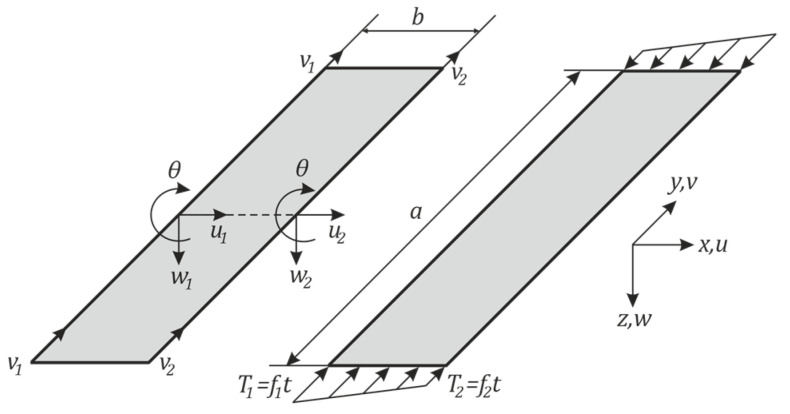
Finite strip Lo2 variant.

The possibilities of the finite strip method were used to solve the given problem. At present, this method does not have such an extensive presence among the commercial computing software currently available on the market, but the simple nature of the method allows it to be programmed in standard programming languages i.e., MATLAB^®^. For the current study of the loss of stability of the cylindrical shell with elliptical cross-section, a lower order strip—Lo2 was used, Figure 8. This type of finite strip is characterized in that each of its nodal lines can move freely in the direction of the z-axis and rotate about the y-axis.

This specific property of the strip results in a pair of coordinate systems for each nodal line and four coordinate systems for one strip. The appropriate displacement function can be written as

(5) $$w=N\;q=\overset{r}{\underset{m=1}{\sum }}N_{m}q_{m}=\overset{r}{\underset{m=1}{\sum }}Y_{m}\left[C_{1}C_{2}\right]q_{m}$$

where, $Y_{m}$ represents polynomial function and its shape is determined by series and based on end condition of strip. $Y_{m}$ is defined in lit. [34] for Lo2 strip variant.

$C_{1},\;C_{2}$ can be defined as

(6) $$C_{1}=\left[\left(1-3{\bar{x}}^{2}+2{\bar{x}}^{3}\right)\;x\left(1-2\bar{x}+{\bar{x}}^{2}\right)\right]$$

(7) $$C_{2}=\left[\left(3{\bar{x}}^{2}-2{\bar{x}}^{3}\right)\;x\left({\bar{x}}^{2}-\bar{x}\right)\right]$$

where $\bar{x}=x/b$, b is the thickness of the strip and

(8) $$q_{m}^{T}={\left(w_{1m}\theta_{1m}w_{2m}\theta_{2m}\right)}^{T}$$

are the displacement and rotation parameters in the two longitudinal edges of the strip for the mth order of sum [34,35]. The above functions ensure the compatibility of displacement values as well as their first partial derivatives at the strip interface, from which a convergent solution can be expected. Equations (6)–(10) for $Y_{m}$ (summation part of displacement functions) can be used for static analysis, although all five basic sum functions must be used for dynamic analyses. A comprehensive derivation of the relationships for the Lo2 strip is given in [34,35,36].

(9) $$C_{1}=\left(1-\bar{x}\right);\;C_{2}=\bar{x},$$

(10) $$C_{1}=\left[\left(1-10{\bar{x}}^{3}+15{\bar{x}}^{4}-6{\bar{x}}^{5}\right)\;x\left(1-6{\bar{x}}^{2}+8{\bar{x}}^{3}-3{\bar{x}}^{4}\right)\;x^{2}\left(0.5\bar{x}-1.5\bar{x}+1.5{\bar{x}}^{2}-0.5{\bar{x}}^{3}\right)\right],\;C_{2}=\left[\left(10{\bar{x}}^{3}-15{\bar{x}}^{4}+6{\bar{x}}^{5}\right)\;x\left(-4{\bar{x}}^{2}+7{\bar{x}}^{3}-3{\bar{x}}^{4}\right)\;x^{2}\left(0.5\bar{x}-{\bar{x}}^{2}+0.5{\bar{x}}^{3}\right)\right].$$

After deriving individual stiffness matrices for a specific case of shell boundary condition, it is necessary to assemble individual blocks of stiffness matrices. In the first block of matrices, the matrices are referred to as the elasticity matrix $k_{e}$. Each such block of matrices consists of two components. These components are referred to as the $k_{\mathrm{em}}^{mn}$ bending and $k_{\mathrm{eb}}^{mn}$ membrane stress sub-matrices. Both sub matrices contain corresponding longitudinal expressions for the sinusoidal half wave m and n [36,37]. The constructed block of stiffness matrix has the form

(11) $$k_{e}^{mn}=\left[\begin{array}{cc} k_{\mathrm{em}}^{mn} & 0\\ 0 & k_{\mathrm{eb}}^{mn} \end{array}\right]$$

where the sub matrices are determined

$$k_{\mathrm{em}}=\overset{}{\underset{V}{\int }}B_{m}^{T}\;D\;B_{m}dV$$

(12) $$k_{\mathrm{eb}}=\overset{}{\underset{V}{\int }}B_{b}^{T}\;D\;B_{b}dV$$

The second type is matrices of geometric stiffness $k_{g}$. Similarly, the elastic matrix $k_{e}$,$\;k_{g}$ contain expressions for m and n, and it corresponds to one block of the geometric stiffness matrix, so for $k_{g}$ it is possible to write

(13) $$k_{g}^{mn}=\left[\begin{array}{cc} k_{\mathrm{gM}}^{mn} & 0\\ 0 & k_{\mathrm{gB}}^{mn} \end{array}\right]$$

where 0 is the matrix containing zero elements and its size is 4×4.

Each matrix constructed in this way (11) and (13) takes the dimension 8×8 and in [34,38] the derived elements for each matrix are listed.

The next step is to transform the individual elements into a global coordinate system. For this purpose serves the transformation matrix whose form is written as

(14) $$\Gamma =\left[\begin{array}{cc} r & 0\\ 0 & r \end{array}\right]$$

where **0** represents a zero matrix of dimension 4×4 and r is defined as

(15) $$r=\left[\begin{array}{cccc} a*cos\beta & 0 & -(b*sin\beta ) & 0\\ 0 & 1 & 0 & 0\\ b*sin\beta & 0 & a*cos\beta & 0\\ 0 & 0 & 0 & 1 \end{array}\right]$$

where a with b represents the ellipse arms.

For correct transformation of all elements and its coordinate systems is used the form

(16) $$K^{e}=\Gamma^{e}{}^{T}\;k^{e}\;\Gamma^{e}$$

where $K^{e}$ represents the eth stiffness matrix in global coordinates, $\Gamma^{e}$ represents the transformation matrix of the eth, and $k^{e}$ represents eth matrix of stiffness in the local coordinate system. Subsequently, if all stiffness matrices are correctly transformed into a global coordinate system, it is possible to assemble these matrices and thus create the main stiffness matrix K for the investigated structure, which in our case represents a thin-walled cylindrical shell. The other step of the solution is the calculation of eigenvalues and eigenvectors with

(17) $$\det\left[K+\lambda K_{\sigma }{}_{\mathrm{ref}}\right]=0$$

## 3. Proposed Elliptical Cross-Section Shapes and Its Solid Mechanical Fixtures, Results of Experimental and Numerical Measurements

Based on Formula (1), the five values of the eccentricity, which form an elliptical shape, were determined. The values of the eccentricity magnitude together with the dimensions of the individual ellipses are listed in Table 1. It should be noted that the ellipses shown in Table 1 are sized to fit the needs of the table and are for illustration only.

**Figure 9:**
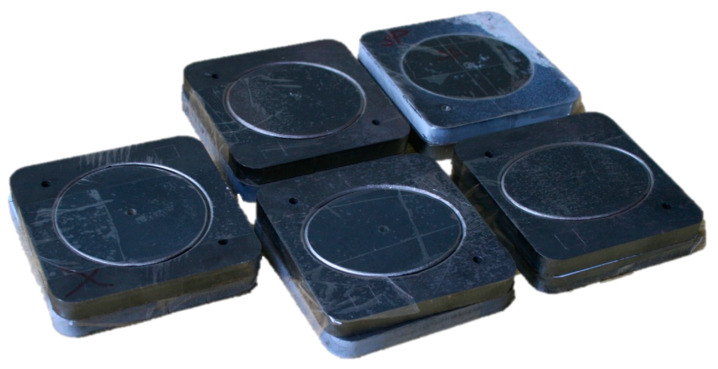
Manufactured variants of mechanical fixtures.

The primary task of the mechanical fixtures is to provide the required elliptical cross-section of the test specimen over its entire length. The secondary task is to properly stabilize the specimen in the test device. Standard structural steel was used to make fixtures and the fixture were made by laser cutting technology. Due to used technology, a continuous thickness of cut was achieved over the entire height of the fixtures, while manufacturing tolerances allow us to consider that fixture produced in this way form a fixed-fixed boundary condition. Figure 9 shows the individual variants of the produced fixtures.

### 3.1. Result of Dimensional Measurement for Surface Wall Thickness and Specimen Width Used for Static Tensile Test

Section 3.1 deals with the evaluation of the wall thickness of the test specimens and the control measured width of the produced specimen for the tensile test (geometric control of the functional dimensions)—specimens that were out of tolerance were not used for the tensile test [39,40,41,42,43].

**Figure 10:**
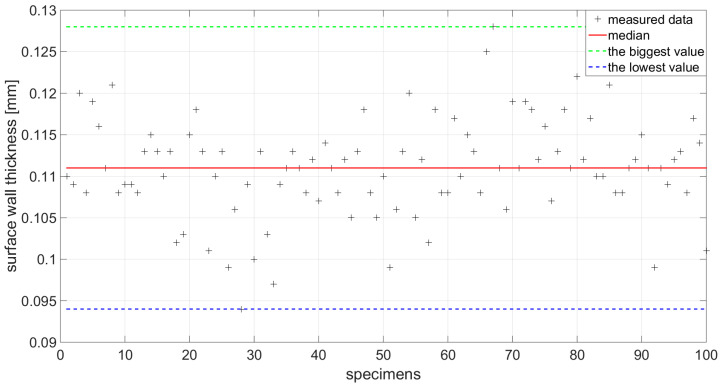
Measured values of wall thickness.

**Figure 11:**
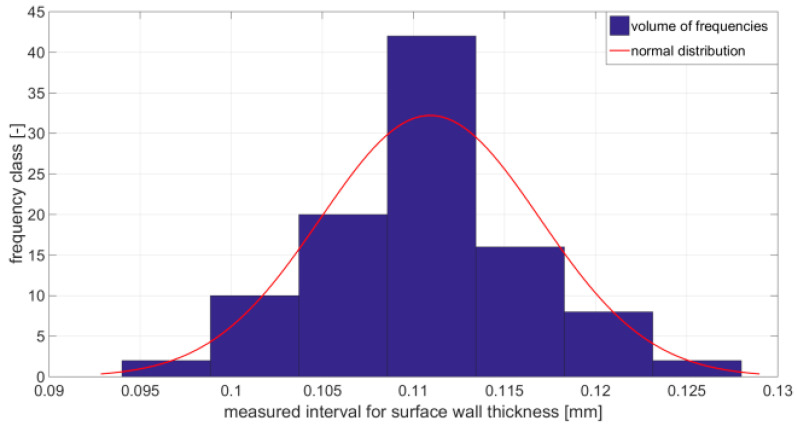
Constructed histogram based on measured values shows the constructed histogram and in Table 2 the measured quantities are given.

The first step is to divide the produced test specimen into the correct class in terms of length. Before the application of Equation (2), it was necessary to determine the average thickness of the shell. About 100 randomly selected cans have been allocated for this task. Measurement of the thickness of the shell surface was made using a micrometer. The measured values were processed using standard statistical methods. Figure 10 shows the scattering of the shell thicknesses of the individual samples. Based on measured values is the constructed histogram (Figure 11).

Thus, by applying Equation (2), using μ=0.33 [26], the value of the shape factor was calculated as Z=2600, since relation (4) applies, the test specimens produced can be considered as a class of medium and long length shells.

**Figure 12:**
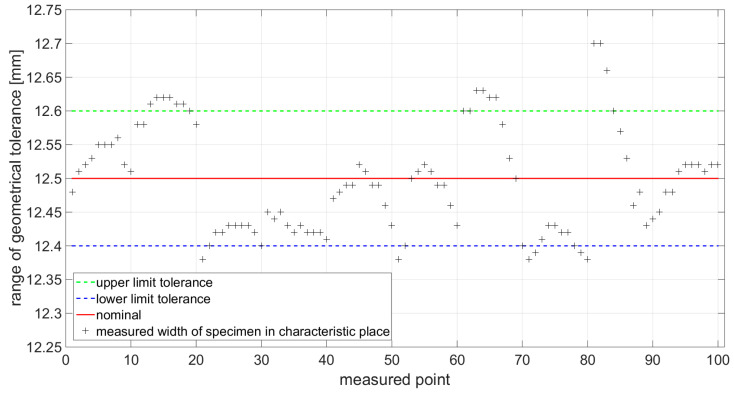
Measured values for specimens with 0° orientation of the material fibers.

**Figure 13:**
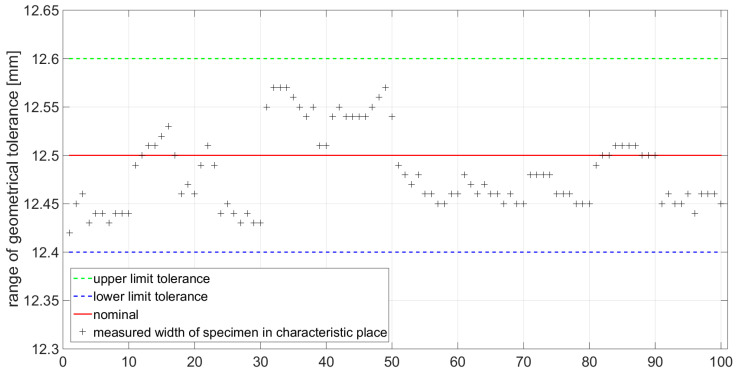
Measured values for specimens with 90° orientation of the material fibers.

The functional dimension defined by the standard [33] was measured at 10 locations at precisely defined points as shown in Figure 7. This measurement was performed to determine if the cutting conditions of the production technology are set correctly. In the case of non-compliance with the dimensional tolerance, the specimens produced cannot be used for the static tensile test. In Figure 12 and Figure 13, the measured values are displayed and measured points from 1 to 10 represent measured points on first specimen, 11 to 20 represent measured points on second specimen, 21 to 30 represent measured points on third specimen, and etc.

Statistically processed and evaluated data are given in Table 3.

### 3.2. Experimental Results of Critical Force Measurement

**Figure 14:**
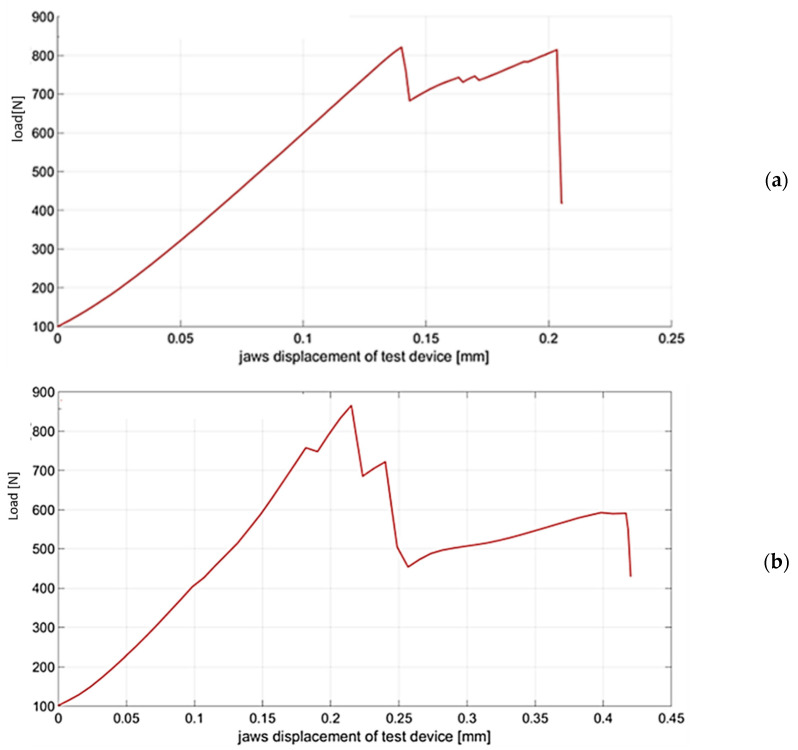

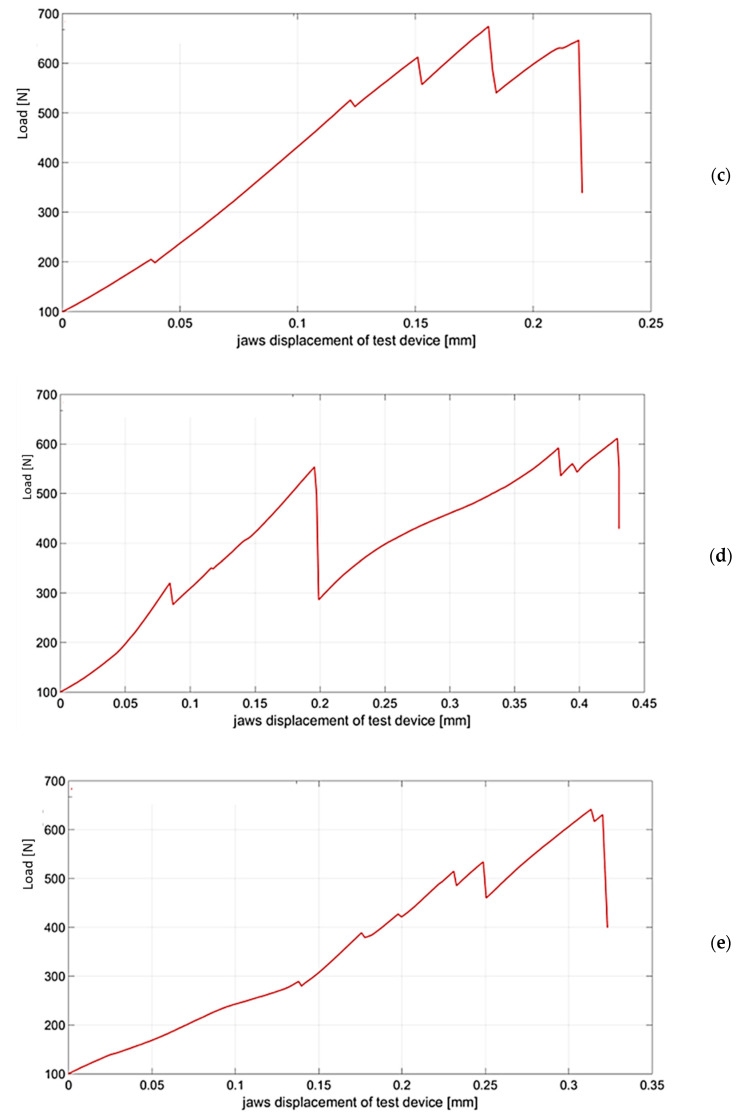
Measurement of critical force $F_{cr}$: (**a**) e = 0; (**b**) e = 0.05; (**c**) e = 0.1; (**d**) e = 0.15; (**e**) ***e*** = **0.20**.

For the needs of experimental measurement and setting of parameters of production technology, approx. 200 test pieces were produced. In order to be able to process and evaluate the critical force measurement performed, it was necessary to repeat the measurement 20 times for each variant of the elliptical cross-sectional area of the test sample. Figure 14 shows randomly selected critical force measurements for the individual elliptical variants of the cross-sectional areas of the test specimens.

**Figure 15:**
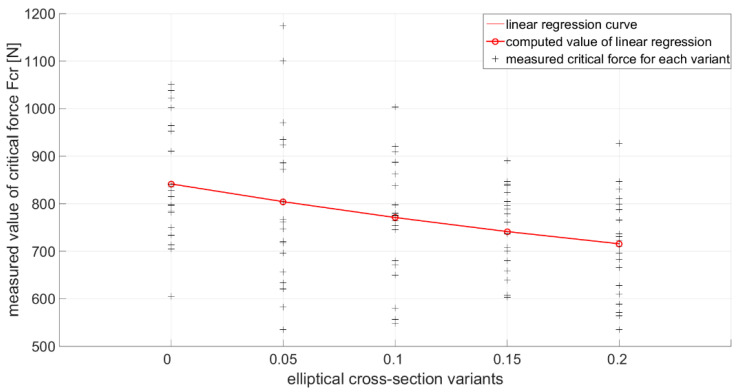
Measurement of critical force $F_{cr}$ for each variant of e and decreasing levels of critical force determined by linear regression.

Figure 15 shows the measured values of the critical force level and shows the resulting decreasing levels of critical force determined by linear regression. In Table 4 the processed measurement data are written.

### 3.3. Result of Static Tensile Test

**Figure 16:**
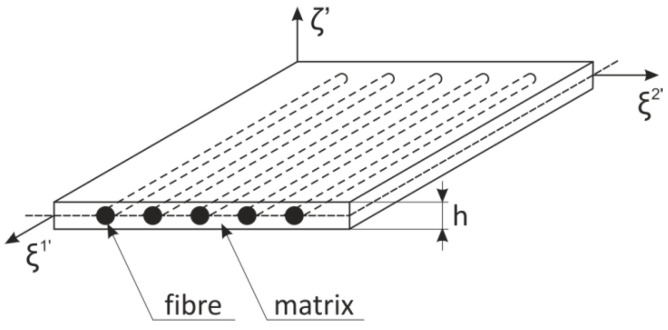
Composite with defined UD–layer.

The task of the static tensile test was to ensure the basic material parameters necessary for the input data of the numerical computation. In order to be able to correctly evaluate the data, it was necessary to repeat the measurement under identical state conditions. As stated in the valid technical standard [33], the specified minimum number for verification measurements is to repeat the measurement using at least 10 new samples. The observed basic material parameters are considered to be the Young’s modulus of elasticity E, the modulus of elasticity in shear of the material G, and the Poisson’s ratio μ. Due to the extensive plastic deformations by which the material of the test specimen is adversely affected, it was not possible to determine the Poisson’s ratio on the basis of the static tensile test. This value was chosen from the literature [26]. The evaluation of the material parameters is based on the assumption that the examined sample, with respect to its geometric dimensions, width to thickness ratio, can in some respects represent one of the layers of composite with unidirectional defined fibers in the material matrix, Figure 16. Thus, the assumed anisotropy of the material, caused as a result of the deep drawing production technology, is simplified for the case of the properties of the orthotropic material.

By introducing a new designation, for $E_{0}=E_{1}$, $E_{90}=E_{2}$ and $\mu =\mu_{12}$, the determination of individual material parameters is approached. The value of $\mu_{21}$ is defined by the relation

(18) $$\mu_{21}=\mu_{12}\frac{E_{1}}{E_{2}}$$

The shear modulus of elasticity in the direction of $G_{12}$, is defined as

(19) $$G_{12}=\frac{\sqrt{E_{1}E_{2}}}{2\left(1+\sqrt{\mu_{12}\mu_{21}}\right)}$$

Plane elasticity tensor of fourth order for a UD-layer in the $\xi^{1\prime },\;\xi^{2\prime }$ coordinate system is defined as

(20) $$E^{\alpha \beta \gamma \delta }=\left[\begin{array}{ccc} E^{1\prime 1\prime 1\prime 1\prime } & E^{1\prime 1\prime 2\prime 2\prime } & E^{1\prime 1\prime 1\prime 2\prime }\\ E^{2\prime 2\prime 1\prime 1\prime } & E^{2\prime 2\prime 2\prime 2\prime } & E^{2\prime 2\prime 1\prime 2\prime }\\ E^{1\prime 2\prime 1\prime 1\prime } & E^{1\prime 2\prime 2\prime 2\prime } & E^{1\prime 2\prime 1\prime 2\prime } \end{array}\right]=\left[\begin{array}{ccc} \frac{E_{1}}{1-\mu_{12}\mu_{21}} & \frac{\mu_{21}E_{1}}{1-\mu_{12}\mu_{21}} & 0\\ \frac{\mu_{12}E_{2}}{1-\mu_{12}\mu_{21}} & \frac{E_{2}}{1-\mu_{12}\mu_{21}} & 0\\ 0 & 0 & G_{12} \end{array}\right]$$

By solving the relation (19) and (20), the values are obtained, which are in Table 5.

The standard deviation for measured modulus G in the axial orientation is 2.048 GPa and in the radial orientation is 0.479 GPa.

### 3.4. Computed Numerical Solutions

In the numerical solution of the loss of stability, the finite element method was also used, to verify the computed values by the finite strip method, and to draw the corresponding collapsed shapes, for each examined cross-section of the test specimens. To verify the calculated values of the finite strip method, the commercial software package NX-Nastran was used (FEM). The accuracy of the calculation of the finite strip method is achieved by the increase of the sinusoidal half-waves. When using the count of approx. 87 sinusoidal half-waves, the calculated value of the critical force for each variant of the test sample changed only at 4 or 5 decimal places. Thus, the solution can be considered convergent for the respective number of sinusoidal half-waves. In the Table 6, the calculated values of the critical forces determined by both numerical methods and the percentage difference in the results are written. It should be noted that both methods had identical material input data and boundary conditions.

**Figure 17:**
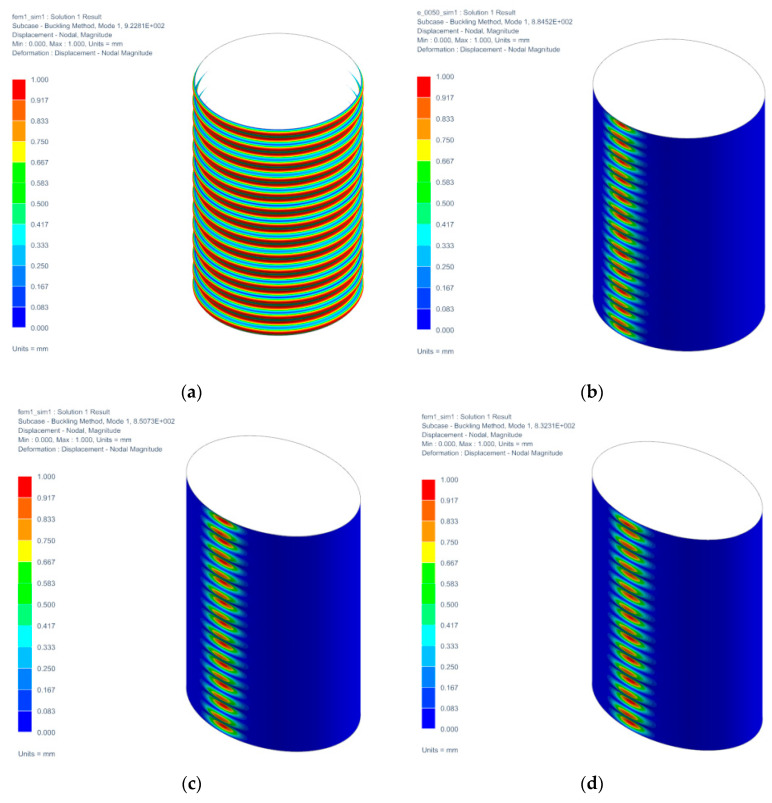

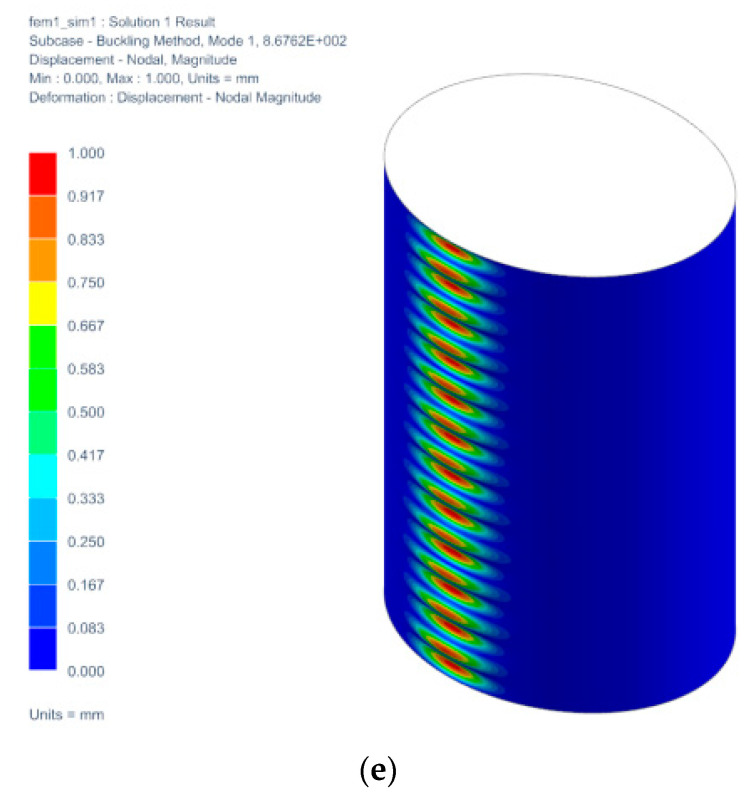
Collapsed shapes for variants: (**a**) e = 0; (**b**) e = 0.05; (**c**) e = 0.1; (**d**) e = 0.15; (**e**) e = 0.20.

In the following Figure 17 are shown the collapsed shapes for the individual variants of the test specimen.

## 4. Discussion

One of the main positive properties of thin-walled shell elements, compared to other thin-walled elements, is the ability to carry higher operating loads. From the point of view of the safety of the experiment, together with the lack of suitable elliptical cylindrical profiles currently on the market, it was decided to produce a test specimen from a can intended for storing various carbonated beverages. Based on the relationship describing the division of a cylindrical shell in terms of its length into the correct class, it showed that during the experimental measurement, certain induced properties of these types of shells were assumed. One of the negative properties, which manifested itself in all investigated variants with an elliptical cross-sectional area and a reference sample, was the establishment of a local loss of stability of the specimen surface [44,45,46,47,48]. This was manifested by the fact that the individual samples collapsed at the minimum applied compressive load. The following are considered to be circumstances which have caused a local loss of stability of the test specimens:

- Geometric deviations of the test specimen surface,
- Geometric deviations of the mechanical fixtures,
- Incorrect placed of the test specimen in the mechanical fixtures,
- Inconsistency of the manufacturing tolerances of the roundness of the casing of the test specimen and the mechanical fixtures,
- Hidden material damage and residual stresses caused by the can manufacturing process,
- Unknown impact.

The test specimens which collapsed did not enter the evaluation process. Another negative factor that was demonstrated was the large variance of the measured critical force levels $F_{cr}$. Using a mechanical fixture, where the size of the elliptical cross-sectional area was determined by e=0.05 the largest variance of the measured critical force levels was recorded. In contrast, when the cross-sectional area was controlled by an eccentricity value of e=0.15, the narrowest variance of the critical force levels was recorded. The presumed cause, which causes such a significant scattering of critical force levels in the case of only one fixture, is considered to be quality and finishing of mechanical fixtures. In the Table 7 are listed the minimum, maximum, and average values together with the values determined by the linear regression.

Table 8 describes the achieved solutions for determining the levels of critical forces by individual methods of solution.

**Figure 18:**
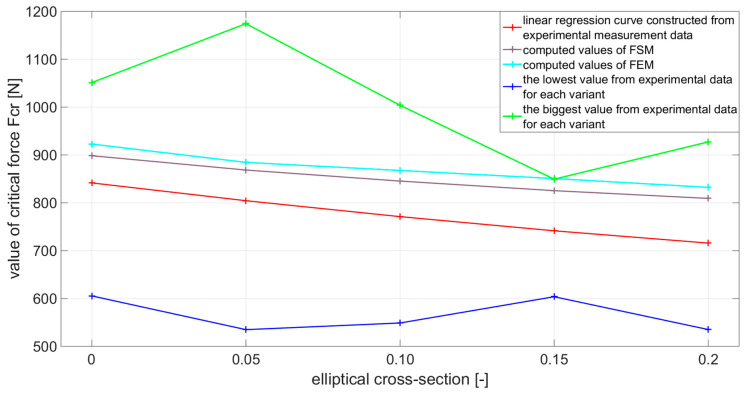
Plotted individual levels of critical force based on Table 8.

Based on Table 8, these conclusions can be drawn. By increasing the value of the eccentricity, which creates an elliptical shape from a circular cross-sectional shape, a decrease is caused in the achieved level of critical force compared to the reference sample. This decrease between the individual variants of eccentricity is about 5%, and the maximum difference is up to 15%, if the reference sample e=0 is compared with the variant e=0.2. The difference between the computed values of the critical force using the finite strip method and the finite element method did not exceed the limit value of 5%. Therefore, the computed values of both methods can be considered correct. In the subsequent comparison between the values of the finite strip method and experimental measurement, the largest difference was approx. 14%. Thus, it is possible to consider the numerical solution to be applicable to the solved problem and corresponding to the solution using the experimental method. In the Figure 18, linear regression values were chosen to plot the decreasing tendency of the critical force due to the increasing value of the elliptical cross-sectional size of the sample [49,50,51,52]. The calculated values and % differences between the individual methods are given in Table 8.

## 5. Conclusions

In the introduction, the article deals with a historical overview of the solution of the problem of loss of stability of thin-walled cylindrical shell elements with an elliptical cross-sectional area. Subsequently, the concept of loss of stability for shell elements and a proposal for solving a given problem are presented in a following second chapter named theoretical background of shell stability and proposed solution of solving stability problem. For the experimental measurement of critical forces, it was necessary to design a suitable test specimen, together with the design of the mechanical fixtures, which will ensure a constant circular and elliptical shape of the casing along its entire length. The size of the ellipses according to which said preparations were made was determined by means of eccentricity. A total of 5 variants were designed in this way, of which one variant represented a reference sample with a circular cross-section. For the need of input material data, both used numerical computation programs, a static tensile test was performed, for which the samples were directly produced from a can blank. In this way, the most accurate material characteristics of the investigated aluminum alloy were ensured. During the experimental measurement, certain characteristic properties of the used shell elements were manifested, which were mentioned in the evaluation part of the article (4. Discussion). The accuracy of the achieved numerical solution of the loss of stability was directly affected by the state of the material, which results in the difference of the calculated values to the values determined experimentally.

It is an indisputable fact that experimental measurements of critical force are critical in the practice of engineering. Practice is the criterion of truth. Indeed, in determining the critical force, there are many factors that can be decisive for its resulting value, which can vary considerably with subtle changes in parameters. Obviously, this type of problem is extremely sensitive to changes in dimensions, material properties, local geometric imperfections, material defects, or boundary conditions. In fact, different types of nonlinearities related to material, geometry, or boundary conditions may be present, which are important not only for the structure as a whole, but especially in certain local parts. From this point of view, this paper is only an approximation in the part related to numerical procedures and does not allow to capture the phenomena related to the ongoing deformation processes of the structure, the so-called postbuckling.

## Figures and Tables

**Table 1:** Proposed elliptical shapes.

Value of e	Ellipse Shape	Dimension of a, b [mm]
e=0	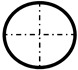	a=33 b=33
e=0.05	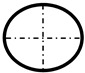	a=34.57 b=31.35
e=0.1	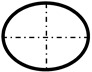	a=35.9 b=29.7
e=0.15	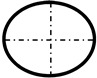	a=37.29 b=28.05
e=0.2	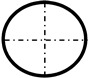	a=38.48 b=26.4

**Table 2:** Processed and evaluated measured quantities.

Standard deviation [mm]	0.006014595
Arithmetic mean [mm]	0.111
Median [mm]	0.111
The biggest value [mm]	0.128
The lowest value [mm]	0.094

**Table 3:** Processed data for measuring the functional dimension of the sample width.

Specimens with Axial Orientation of the Material Fibers		Specimens with Radial Orientation of the Material Fibers	
Standard deviation [mm]	0.0302	Standard deviation [mm]	0.0079
Arithmetic mean [mm]	12.496	Arithmetic mean [mm]	12.483
Median [mm]	12.503	Median [mm]	12.460
The lowest measured value [mm]	12.38	The lowest measured value [mm]	12.43
The biggest measured value [mm]	12.70	The biggest measured value [mm]	12.57

**Table 4:** Processed data from the measurement of critical force levels $F_{cr}$.

Statistical Parameters	Standard Deviation [N]	Arithmetic Mean [N]	Median [N]
Ref. variant e=0	120.2447	805.891	783.580
Variant e=0.05	150.7167	799.594	773.765
Variant e=0.1	112.8161	780.684	754.933
Variant e=0.15	84.3076	743.717	736.544
Variant e=0.2	103.1899	687.421	688.285

**Table 5:** Statistically processed and evaluated material characteristics of test specimens.

Specimens with Axial Orientation of the Material Fibers _1_			Specimens with Radial Orientation of the Material Fibers _2_		
$E_{1}$ [GPa]	$G_{12}$ [MPa]	$\mu_{12}$ [-]	$E_{2}$ [GPa]	$G_{21}$ [MPa]	$\mu_{21}$ [-]
20.53	5632	0.33	12.51	5632	0.54

**Table 6:** Computed values of critical forces by FSM and FEM.

Variant	$\mathrm{FSM}\;F_{cr}\;[N]$	$\mathrm{FEM}\;F_{cr}\;[N]$	% Difference
Ref. e=0	898.4	922.8	2.6
e=0.05	868.5	884.5	1.8
e=0.1	845.4	867.6	2.5
e=0.15	825.4	850.7	2.9
e=0.2	809.2	832.3	2.7

**Table 7:** Processed values of the determined critical force by experimental measurement.

Variant	$\mathrm{Minimal}\;\mathrm{Measured}\;F_{cr}\;[N]$	$\mathrm{Maximal}\;\mathrm{Measured}\;F_{cr}\;[N]$	^1^ Linear Regression of $F_{cr}\;[N]$	$\mathrm{Arithmetic}\;\mathrm{Mean}\;F_{cr}\;[N]$	% Difference between ^1^ and ^2^
Ref. e=0	605.4	1051.3	841.6	805.8	4.4
e=0.05	534.9	1285.5	804.4	799.5	0.6
e=0.1	548.8	1003.8	771.1	780.6	1.2
e=0.15	603.7	849.4	741.5	743.7	0.3
e=0.2	535.1	927.3	715.8	687.4	4.2

**Table 8:** Determining the levels of critical forces by individual methods of solution.

Variant	Linear Regresion ^1^ $F_{cr}\left[N\right]$	^2^ FSM $F_{cr}\;[N]$	^3^ FEM $F_{cr}\;[N]$	% Difference between ^1^ and ^2^	% Difference between ^2^ and ^3^
Ref. e=0	841.6	898.4	922.8	6.3	2.6
e=0.05	804.4	868.5	884.5	7.3	1.8
e=0.1	771.1	845.4	867.6	8.7	2.5
e=0.15	741.5	825.4	850.7	10.1	2.9
e=0.2	715.8	809.2	832.3	11.5	2.7

## Data Availability

Data are contained within the article.
